# Label-free imaging of bone multiscale porosity and interfaces using third-harmonic generation microscopy

**DOI:** 10.1038/s41598-017-03548-5

**Published:** 2017-06-13

**Authors:** Rachel Genthial, Emmanuel Beaurepaire, Marie-Claire Schanne-Klein, Françoise Peyrin, Delphine Farlay, Cécile Olivier, Yohann Bala, Georges Boivin, Jean-Claude Vial, Delphine Débarre, Aurélien Gourrier

**Affiliations:** 1 Univ. Grenoble Alpes, LIPHY, F-38000 Grenoble, France; 2 CNRS, LIPHY, F-38000 Grenoble, France; 3 LOB, Ecole Polytechnique, CNRS, Inserm, Université Paris-Saclay, F-91120 Palaiseau, France; 4 Université de Lyon, CREATIS, CNRS UMR5220, Inserm U1206, INSA-Lyon, Université Claude Bernard, Lyon 1, France; 5 ESRF, European Synchrotron Radiation Facility, F-38000 Grenoble, France; 6 INSERM, UMR 1033, Univ Lyon, Université Claude Bernard Lyon 1, F-69008 Lyon, France; 7 Université de Lyon, F-69008 Lyon, France

## Abstract

Interfaces provide the structural basis of essential bone functions. In the hierarchical structure of bone tissue, heterogeneities such as porosity or boundaries are found at scales ranging from nanometers to millimeters, all of which contributing to macroscopic properties. To date, however, the complexity or limitations of currently used imaging methods restrict our understanding of this functional integration. Here we address this issue using label-free third-harmonic generation (THG) microscopy. We find that the porous lacuno-canalicular network (LCN), revealing the geometry of osteocytes in the bone matrix, can be directly visualized in 3D with submicron precision over millimetric fields of view compatible with histology. THG also reveals interfaces delineating volumes formed at successive remodeling stages. Finally, we show that the structure of the LCN can be analyzed in relation with that of the extracellular matrix and larger-scale structures by simultaneously recording THG and second-harmonic generation (SHG) signals relating to the collagen organization.

## Introduction

Bones are complex systems from a material science point of view. They are characterized by a high degree of structural hierarchy, a mineral/organic nano-composite structure and they exhibit heterogeneity at all structural length scales^1^. As a result, a combination of physical, chemical and biological parameters needs to be considered for functional analysis^2–4^. This structural complexity stems from multiple functional requirements, including biomechanical integrity, red blood cell production and calcium regulation, which are intimately connected to bone cellular activity and to the associated biological fluids circulation.

**Figure 1:**
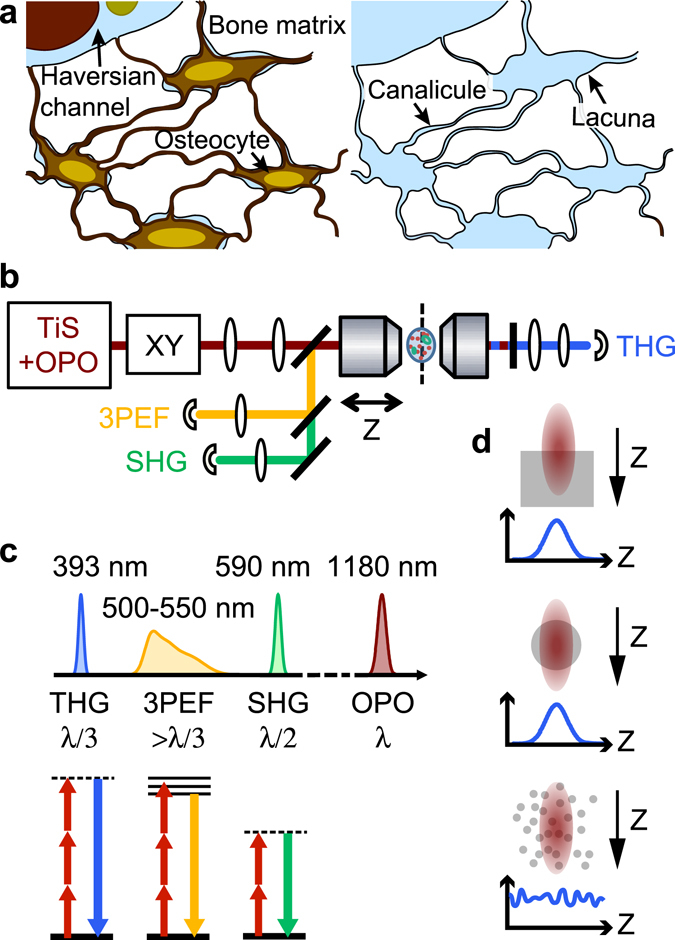
Lacuno-canalicular network and principles of THG-SHG-3PEF microscopy. (**a**) Schematic view of bone structure at the micrometric scale. Left, the network of interconnected osteocytes is embedded in the bone matrix and connected to Haversian channels containing nerves and blood vessels. Right, once the cells are removed, their previous location forms a network of porosities consisting of lacunae and canaliculi, forming the LCN. (**b**) Scheme of the microscope used for nonlinear imaging. An optical parametric oscillator (OPO) pumped by a titanium-sapphire laser (Ti:S) is focused and scanned inside the sample using galvanometric mirrors (XY) and a high-NA objective. Third harmonic generation (THG), second harmonic generation (SHG) and three-photon excited fluorescence (3PEF) signals are excited simultaneously and detected on photomultiplier tubes after spectral separation. 3PEF and SHG are epidetected while THG is collected in transmission through the sample. (**c**) Spectral representation (top) of the signals created by OPO excitation at λ = 1180 nm. THG (resp. SHG) is generated at a third (resp. half) of the excitation wavelength, while 3PEF is emitted in a broad range of wavelengths longer than λ/3. Bottom, corresponding simplified Jablonski diagrams. Dashed lines, virtual states. (**d**) THG signal for various sample geometries relevant for bone imaging. Interfaces (top) and heterogeneities down to a fraction of the size of the focal volume give rise to a localized signal, while a uniform medium produces no signal. Smaller size, randomly distributed heterogeneities create a non-zero, fluctuating signal.

In these different processes, interfaces play a central role as they both influence the mechanical properties of the tissue^5^ and control the cell/material interaction. In particular, multiscale porosity provides a large internal exchange surface: at the macroscopic level, the bone envelope defines a closed interface between bone marrow and the surrounding connective tissue. At a smaller scale, the vascular channels permeating the cortical shell constitute a second level of interface within bone, with typical channel diameters in the range 0.1–1 mm. It is also connected to the lacuno-canalicular porosity (~0.2–1 µm) which hosts the dense, interconnected network of cells embedded in the bone tissue, the osteocytes^6^ (Fig. 1a). Finally, the interstitial fluid is also thought to permeate bone tissue through a nanoporosity network (~1–10 nm)^7^.

In addition, other forms of interfaces are present in bone tissue as a result of bone formation and remodeling: histological analysis of bone sections allows identifying contiguous volumes with a characteristic length scale of ~100 µm formed at different time intervals, generally referred to as bone structural units (BSU)^8^. Because of the composite nature of bone, the interface between two (or more) BSUs can be identified by a relative change in properties of the associated mineral and/or collagen phases. In human cortical bone, for example, osteons are generally separated from the surrounding tissue by a cement line of less than 5 µm in thickness^9^. Within a BSU, the organic matrix organization can give rise to sharp transitions associated with abrupt changes in collagen orientation over distances of ~5–10 μm^10, 11^. Finally, the surface of the collagen fibrils (~0.1–1 µm) as well as that of the mineral nanocrystals (~5–100 nm) constitute the interfaces of smallest dimension^12, 13^.

All these interfaces, whether between tissue and fluids, mineralized and soft tissue, collagen and mineral, have a profound impact on the physical, chemical and biological properties of bone. There is thus a considerable interest in establishing novel methods for their characterization that would provide specific contrast with appropriate three-dimensional resolution^14^.

In recent years third-harmonic generation (THG) microscopy^15, 16^, a label-free form of multiphoton microscopy, has emerged as a powerful technique for detecting interfaces and heterogeneities in cells and tissues. Lipid-water interfaces^17^, boundaries between collagen domains^18^ and fibers^19^, or cell-derived vesicles^20^, for example, have been imaged *in vivo* or *ex vivo* with submicron resolution. THG is a nonlinear scattering process whereby three (usually infrared) photons are converted into a single (visible) photon with exactly three times the incident energy (Fig. 1b and c). The efficiency of this coherent process is governed by phase matching between the excitation and scattered fields near focus, which results in a particular contrast mechanism: in THG microscopy images, no signal is generally obtained from homogeneous regions, and optical heterogeneities at the micron or submicron scale are highlighted over a dark background (Fig. 1d). This contrast permits structural imaging of living and/or intact tissue^21–23^ with the benefits associated with nonlinear microscopy: three-dimensional sectioning, ability to image within thick samples, and limited photodamage^24^. Its straightforward combination with second-harmonic generation (SHG) and two- and three- photon excited fluorescence (Fig. 1b) has further extended its applications, ranging from the tracking and lineage of cells in developing embryos^25^, to their interaction with the surrounding extracellular matrix and the study of tissue-guided migration^20^, or of hemodynamic parameters^26^.

In this paper, we demonstrate the use of THG microscopy for label-free, multiscale imaging of interfaces in bone. Extending beyond the pioneering work of Oron *et al*.^27^, we show that THG provides detailed quantitative information on bone porosity and, in particular, that the achievable resolution is sufficient to image the lacuno-canalicular network (LCN) from the sub-micrometer scale up to the organ level, even in decalcified or resin-embedded samples for which fluorescence staining cannot be used. We show that the boundaries of the BSUs can also be delineated in THG images providing new insight into bone formation that is not usually accessible using optical microscopy. Exploiting the possibility to simultaneously acquire the second-harmonic generation signal specific to collagen fibrils, we then analyze the THG observations in the light of the collagen matrix organization. Finally, we show that such structural information can be combined with functional fluorescence imaging, providing complementary information to decipher the complex processes at play during bone formation and remodeling.

## Results

### Label-free THG imaging reveals the cellular porosity network in bone

**Figure 2:**
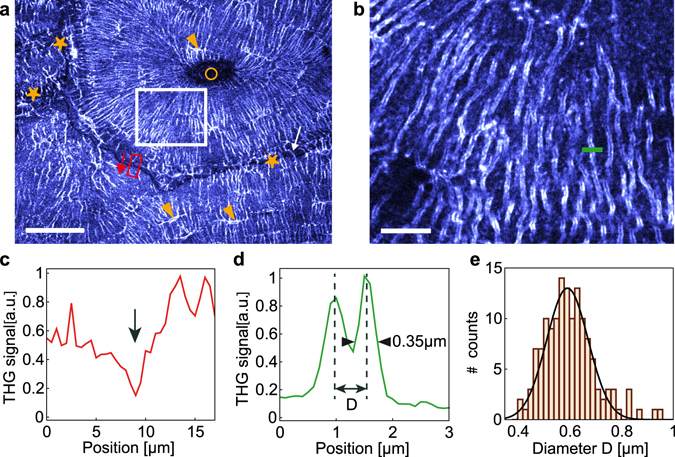
THG reveals interfaces in cortical bone. (**a**) THG image of a transverse section of a bovine femur at a depth of 10 µm within the sample, revealing the LCN (lacunae indicated with arrowheads) and the cement line of the osteons (stars). Circle, Haversian channel. The white arrow shows a canalicule crossing the cement line. Scale bar, 50 µm. (**b**) Zoom on the white box shown in (**a**), revealing the two interfaces of the canaliculi. Scale bar, 10 µm. (**c**) Profile along the red box in (**a**), illustrating the reduced signal at the cement line (arrow). (**d**) Profile across a canalicule along the green line in b providing an estimate for the canalicule diameter D and for the lateral resolution. (**e**) Distribution of canalicule diameters D estimated from THG images in the volume shown in Supplementary Movie 1 including image (**b**).

Typical THG images of cortical bone structures are shown in Fig. 2 on a transverse section of a bovine femur. The highest intensity values reveal features that closely resemble the structure of the osteocyte cellular network embedded in the bone matrix. However, due to the sample preparation involving ethanol fixation and dehydration, the signal most likely originates from the associated porosity hosting the osteocytes, the lacuno-canalicular network (LCN). Overall, the lacunae, indicated by arrowheads, appear thin and elongated and the canaliculi seem to be mainly aligned normal to the lacunae. In the osteon, the lacunae are oriented in the tangential direction, while the canaliculi extend radially from the center of the vascular Haversian channel appearing dark on the THG image, as described in previous studies (see, e.g. ref. 28).

Other structures than the LCN are also visible in the THG images. In particular, a non-negligible signal arises from the bone matrix, forming a background to the LCN-induced signal. As evidenced in Fig. 2a and c, this background is not homogeneous and, in particular, drops to lower values at the position of the cement lines (indicated by stars on Fig. 2a) around the osteons. Since the cement line differs in mineral density and organic matrix composition from its surroundings^9^, this indicates that the matrix-induced THG signal varies depending on the tissue structure and/or composition. This simultaneous visualization of the LCN and the cement line therefore provides a direct means to study the network structure at the boundary of the osteons: while it is generally agreed that the canaliculi are connected to the inner vascular channel of the osteons, the existence of canaliculi crossing the cement line is more controversial^28, 29^. Our THG images allow visualizing such connections (e.g. white arrow on Fig. 1a) and quantify their density, which appears much reduced compared to the rest of the tissue (Supplementary Movie S2). This is in good agreement with recent findings showing that the number and density of such connections depend on the age of tissue^30^.

We investigated the achievable spatial resolution of our method using a high-numerical aperture objective (oil immersion, 1.35NA), and found that it is possible to resolve the two vertical edges of the canaliculi in the image plane (Fig. 2b). Typical intensity profiles measured across the canaliculi (Fig. 2d) yield an optical resolution of 0.35 µm (lateral) × 1.35 µm (axial), larger than theoretical values (see Supplementary Table 1). Such lateral resolution is nevertheless sufficient to estimate the diameter of the canaliculi, calculated as the distance between the two edges on the THG image. As an example, we measured manually the diameter of the canaliculi in random positions in a 60 × 60 × 50 μm^3^ volume (Fig. 2b): the average diameter was estimated to be 0.57 µm +/−0.1 µm (Fig. 2e), about twice larger than reported values in mice^31^ but in the range of measured diameters in human femur^32^ and in very good agreement with a previous study on a sheep tibia^33^ (see also^34^ for a summary of published values). This confirms that the resolution provided by THG imaging is sufficient to visualize sub-micrometric details and, thus, might enable dimensional and volume analysis of the LCN. The precision of such quantification should be estimated by comparison with established methods such as electron microscopy^34^.

**Figure 3:**
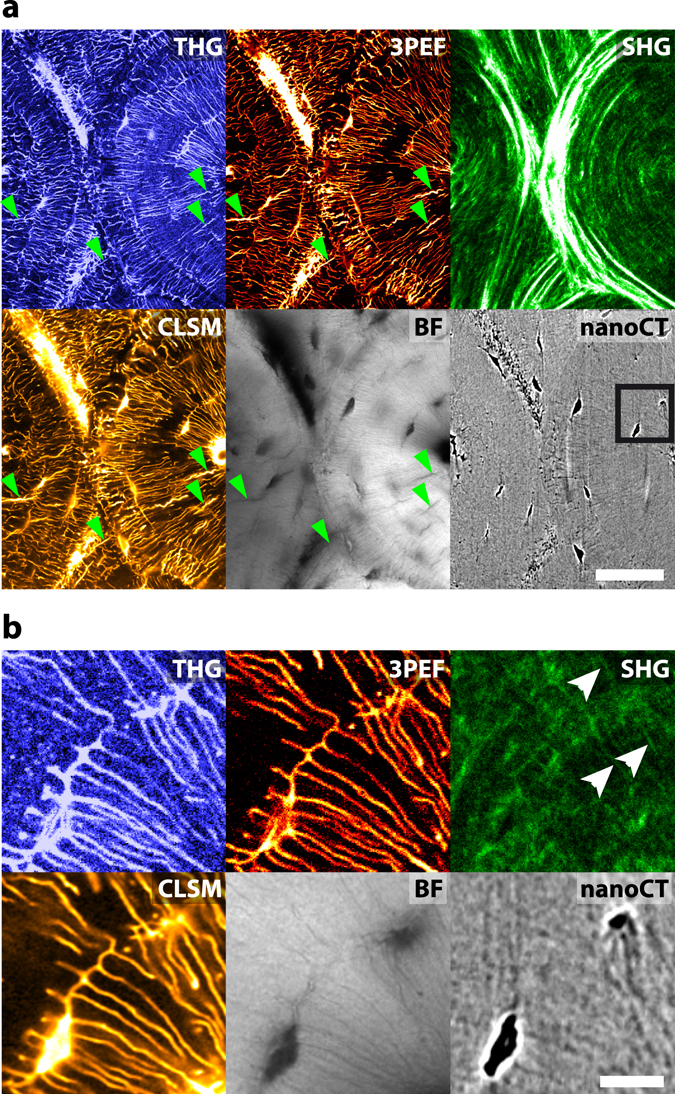
Comparison of different bone imaging modalities. (**a**) Wide field comparison of THG (top left), 3PEF (top middle), SHG (top right), fluorescence confocal (CLSM, bottom left), brightfield transmission (BF, bottom middle) and X-ray nanoCT (bottom right) images acquired on a transverse section of a bovine femur. Some cracks are indicated by green arrowheads in (**a**). Additional cracks are visible on the confocal and brightfield images that were recorded after the nonlinear images. Scale bar, 50 µm. (**b**) Zoom in the black rectangle in (**a**). Collagen fibrils along some of the canaliculi are shown by white arrows on the SHG image. Scale bar, 10 µm. See also Supplementary Movie 3 and 4.

To validate the previous observations, a site-matched comparison was performed with confocal laser scanning fluorescence microscopy (CLSM) and X-ray computed nano-tomography (nanoCT) using cortical bovine bone within a region of interest between two osteons observed in transverse sections (Fig. 3). To correlate the images obtained by THG and confocal fluorescence microscopy, the three-photon excited fluorescence (3PEF) signal of the sample stained with FITC was acquired simultaneously with the THG signal. Here, we used an objective with a slightly reduced numerical aperture (water immersion, 1.2NA) that does not permit to resolve the two edges of the canaliculi in the THG images, thus enabling a more direct comparison with fluorescence. Because THG and 3PEF signals originate from the same excitation volume, those two images are intrinsically colocalized. The correspondence with confocal fluorescence microscopy was, then, performed by registering the 3PEF and confocal images. As expected, both linear and non-linear fluorescence images delineate the LCN with a very good specificity ensured by the staining, although the signal-to-noise ratio of 3PEF image is poorer due to the lower efficiency of this higher-order nonlinear process. The corresponding CT and transmission images confirm that all the lacunae in the optical section are stained and that they appear in the THG image. A direct comparison between fluorescence and THG images confirms that this latter modality also permits visualizing the canaliculi of the LCN (correlation coefficient of the THG image in Fig. 2b with the corresponding fluorescence confocal image: 0.60; with the 3PEF image: 0.66; see methods for details). It should also be noted that, as with fluorescence imaging and nanoCT, other forms of porosity than lacunae and canaliculi, such as cracks (indicated by arrowheads on Fig. 3a), can be observed in THG images.

The downside of the additional signal arising from the bone matrix is a reduction of the contrast of the canaliculi in THG images (canaliculi-to-background ratio 3.4:1) compared to fluorescent images obtained with a specific staining (signal-to-background ratio 9.3:1 for 3PEF, 8.2:1 for confocal). Nevertheless, this contrast compares favorably with that of the nanoCT image resulting from X-ray absorption contrast (1.4:1; see Methods) although this limitation can be mitigated by post-processing^35^. Even though such quantitative comparison is difficult due to the large variability induced by the acquisition parameters for each modality, these numbers illustrate the potential of THG for the characterization of the LCN in unstained samples.

In addition to 3PEF signals, second-harmonic generation (SHG) images were also recorded in the backward (epidetected) direction during THG image acquisition. SHG provides an intrinsic contrast specific to fibrillar collagen, in particular in the bone matrix^36–40^, and hence provides an image of the collagen matrix intrinsically registered with the THG image. The SHG signal depends on the degree of alignment and packing density of the collagen fibrils as well as their relative orientation with respect to the imaging plane and to the polarization of the excitation beam. While polarimetric imaging permits extracting quantitatively the orientation of collagen fibrils^36, 37, 39–44^, in this paper we only recorded images for a single linear polarization, which only provides qualitative information about fibril orientations: high intensities are indicative of dense assemblies of well aligned fibrils with the same polarity, essentially lying in the plane of observation, while a lower signal can result from either (or both) a less-organized collagen matrix or an out-of-plane fibril orientation. The large concentric intensity modulations observed in Fig. 3 are attributed to periodic variations in the out-of-plane orientation of densely packed collagen fibrils, according to previous electron microscopy^45–47^, X-ray scattering^11^, and phase-contrast X-ray imaging^48^ studies. The two osteons can clearly be delimited by the SHG signal which also reveals the lamellar organization of the interstitial tissue at the top and bottom parts of the image. It can be noted that variations in the SHG signal correlate with opposite changes in the background THG signal, albeit with a reduced amplitude (see also Fig. 9e), which confirms that this background THG signal is sensitive to the sub-micrometric organization of the matrix. Interestingly, a faint SHG signal, indicated by the white arrows in the high magnification image (Fig. 3b), reveals the presence of collagen fibrils along some of the canaliculi. This indicates a change in the matrix organization in the immediate vicinity of the LCN. The faintness of this signal suggests that the amount of collagen is limited, which is consistent with observations of an increased mineral content in the vicinity of the canaliculi^49^. This observation illustrates how THG and SHG images provide complementary information on the porosity and tissue organization in the peri-lacunar and peri-canalicular bone.

### 3D visualization of the LCN and segmentation

**Figure 4:**
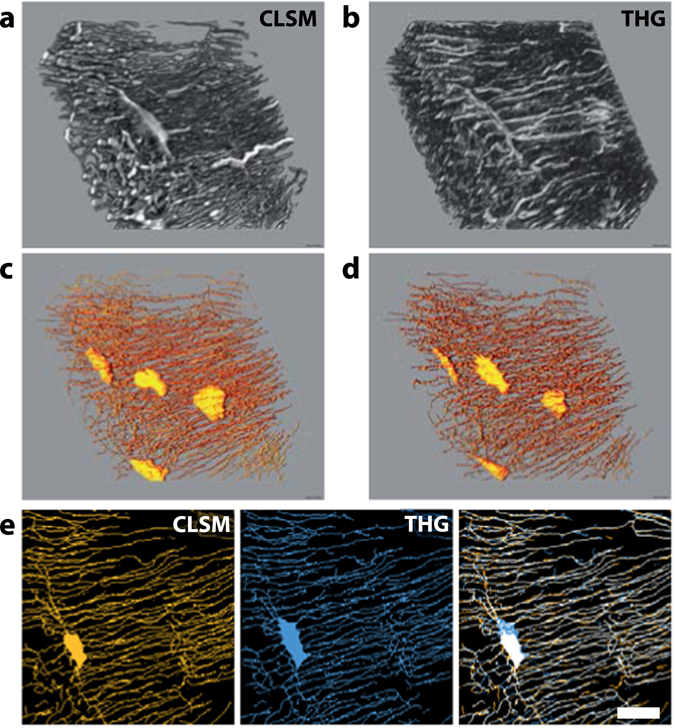
3D segmentation of the LCN. 3D reconstructions of a 50 × 50 × 20 μm^3^ volume of a bovine femur imaged with THG (**a**) and confocal fluorescence (CLSM, **b**). (**c**) (resp. (**d**)), skeletonization of the segmented signal shown in (**a**) (resp. (**b**)). (**e**) Comparison of the LCN skeleton calculated from fluorescence (CLSM, left) and THG (middle) images. The overlay of the two retrieved skeletons (right) highlights the consistency of the results obtained with the two techniques. Scale bar, 10 µm.

The use of THG to characterize the LCN quantitatively is further demonstrated in Fig. 4 which shows the three-dimensional reconstruction of THG and confocal stacks of images, covering a 50 × 50 × 20 μm^3^ volume. These reconstructions obtained without any post-processing of the raw images illustrate the comparable spatial resolution achieved with the two techniques in our imaging conditions. Although the LCN is less contrasted in THG images, the quality of images is sufficient to accurately segment the network (Fig. 4,c and d) up to about 50 µm deep inside the sample. At greater depths, optical aberrations induced by the tissue and, in particular, by the lacunae, reduce the quality of images and the achievable level of signal (see Supplementary Figure 1). A comparison of the segmented LCN using confocal and THG imaging is shown in Fig. 4e using axial projections on 10 µm of the stacks from Fig. 4a and b. While minor changes can be observed in the shape of the canaliculi between the two images, the overall structure of the network is consistent using both techniques (correlation coefficient = 0.57). From such segmentation, quantitative information on the LCN can be extracted such as the density of porosities. Using the diameter of canaliculi determined in the previous subsection, we found that the total porosity represents 3.65% of the imaged volume (3.55% when using the confocal images) and, more specifically, that canaliculi account for 3.55% (respectively 3.4%) of the selected volume, which is in good agreement with similar analyses^34, 50, 51^.

### Multiscale imaging of the LCN

While the penetration depth of THG/SHG imaging is limited, the accessible in-plane field of view is virtually unlimited when performing mosaic acquisitions. We illustrate the potential of such large field imaging for bone structure analysis in two widely used model systems, murine and bovine cortical bone, which constitute two extreme cases in terms of bone formation, cortical thickness and tissue organization.

**Figure 5:**
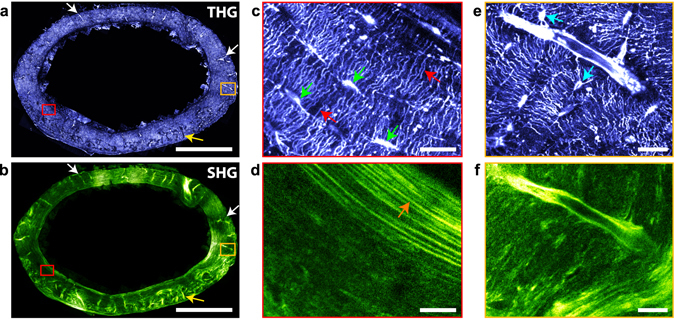
Imaging mouse bone structures at the organ scale. Whole section of a mouse femur imaged a few microns below the sample surface with THG (**a**) and SHG (**b**). Tubular structures of diameter consistent with that of vascular channels (~11 ± 3 µm), appear either with a positive (white arrows) or negative (yellow arrow) contrast in the THG images. Scale bar, 500 µm. Total imaging time, 333 s (including overlaps between images). (**c**) (resp. (**d**)), zoom into the red rectangle area in (**a**) (resp. (**b**)), showing thin and elongated lacunae along the tangential direction, i.e. parallel to the endost and periost (green arrows), canaliculi mainly oriented along the radial direction (red arrows) in the THG image, and a collagen lamellar periodic structure in SHG (orange arrow). Scale bar, 20 µm. (**e**) (resp. (**f**)), zoom into the orange rectangle area in (**a**) (resp. (**b**)), showing a vascular channel, rounder lacunae (blue arrows) and a more disordered collagen structure.

In Fig. 5a and b, the full cortical transverse section of a mouse femur was imaged using 52 stitched images, accounting to a total field of view of 2 × 1.4 mm^2^ with a 200 nm pixel size and a 350 nm lateral resolution (axial resolution is 1.8 µm). As previously described, THG reveals porosities of various sizes in the tissue: tubular structures of diameter consistent with that of vascular channels (~11 ± 3 µm) either appear with a positive (white arrows and Fig. 5e) or negative (yellow arrow) contrast, most probably due to the presence or absence of remaining organic matter in the channel (see also Fig. 7). They are mainly radially oriented and, in most cases, are surrounded by an intense SHG signal which reveals a local change in the collagen structure with dense fibrils aligned along the vessels (Fig. 5b and f). In the close vicinity (~5–10 µm) of the vascular channels, a reduced number of connections can be observed between the adjacent lacunae and the vessels (Fig. 5e). Overall, the lacunae appear to be thin and elongated along the tangential direction (Fig. 5c, green arrows), i.e. parallel to the endost and periost (inner and outer bone surfaces respectively), while the canaliculi are mainly oriented along the radial direction (Fig. 5c, red arrows). This high degree of ordering is more pronounced towards the endost and the periost, where thin SHG modulations reveal a highly ordered collagen organization in a periodic lamellar structure of 4.2 ± 1.4 µm in thickness on average (Fig. 5d, orange arrow). On the contrary, the LCN appears more disordered in the inner part of the cortical shell, with rounder lacunae (Fig. 5e, blue arrows), less oriented canaliculi (Fig. 5e) and a greater variability in the SHG signal, i.e. in the collagen structure (Fig. 5f). Interestingly, we also find a larger-scale variation of the LCN organization in this sample, which seems more regular across the larger cortical thickness of the medial and lateral quadrants and appears more chaotic in the thinner anterior and posterior cortical shells where more vascular channels are observed.

**Figure 6:**
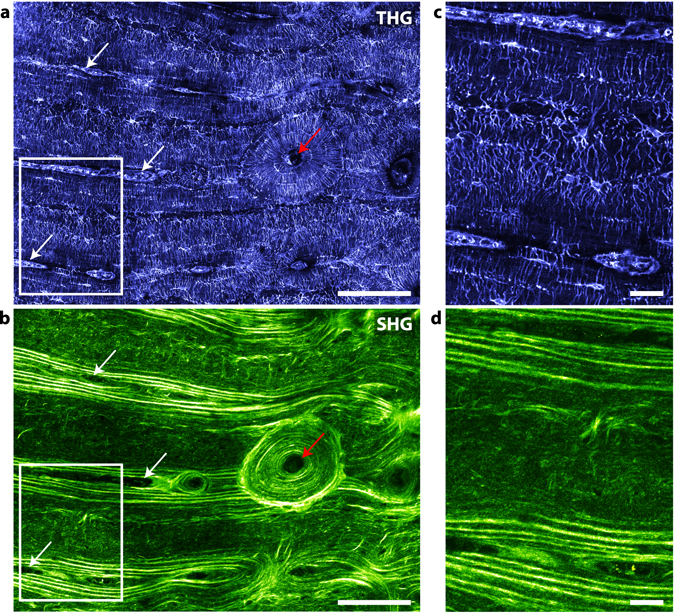
Imaging structural variability in bovine bone. Large-scale image of a transverse section of cortical bovine femur in THG (**a**) and SHG (**b**). This large-scale image includes an osteon with a well-defined radial organization (around the Haversian channel, red arrow), and a surrounding fibrolamellar region. Scale bar, 100 µm. (**c**) (resp. (**d**)), zoom into the rectangle area in (**a**) (resp. (**b**)). At the bottom and top parts of the images, lamellar regions surrounding vascular channels (white arrows in (**a**,**b**)) correspond to well-aligned lacunae and canaliculi, while the fibrous region in the center shows rounder lacunae and more disordered canaliculi. Scale bar, 10 µm.

As a second example, Fig. 6 shows a representative region of interest of the cortical shell of a bovine femur. Again, THG outlines porosities of various sizes in the tissue: the central vascular channels in fibrolamellar regions (white arrows) and in the center of the osteon (red arrows) exhibit punctuate THG signals, attributed to residual organic material, while appearing dark in the SHG image. As in Fig. 2, the dense LCN appears as a well contrasted positive signal. The complementarity between the SHG and THG images is obvious: in the osteon, the THG data reveal that the LCN network is essentially organized along the radial and tangential directions with respect to the center of the osteon (Fig. 6a; see also Figs 2a and 3a), while the corresponding SHG images reveal a concentric organization of collagen with intensity modulations attributed to variations in collagen orientation relative to the imaging plane, as in Fig. 3. The period of the modulations is 5.6 ± 2.2 µm on average (see Supplementary Figure 2). The rest of the SHG image reveals a characteristic collagen organization of fibrolamellar bone which consists of alternating layers of lamellar and woven bone^52^. In lamellar bone, the lamellae are more contrasted than in the osteon, which a period of 4.8 ± 1.2 µm on average (see Supplementary Figure 2), and also correlate with a regular organization of the LCN in which elliptical lacunae and canaliculi are essentially aligned along and normal to the direction of the lamellae, respectively (Fig. 6c and d). In contrast, the woven bone appears much darker in the SHG image, albeit with high intensity fibrous-like features which typically lack long-range correlation. Conversely in this region, the lacunae are found to be more circular and the canaliculi radiate in a more isotropic fashion with a higher degree of disorder.

These two examples illustrate the performance of large-scale THG/SHG imaging, which provides a comprehensive description of the microstructural heterogeneity of bone tissue.

### THG contrast in bone is affected by the sample embedding medium

**Figure 7:**
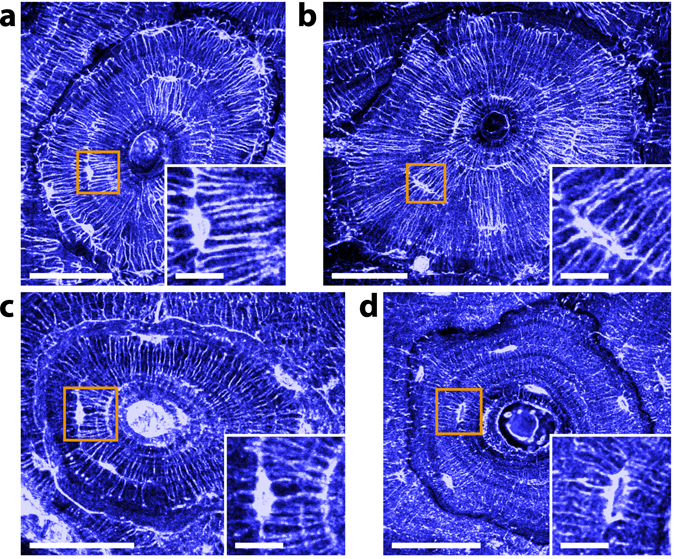
Effect of the sample preparation on THG imaging. THG images of (**a**), dehydrated; (**b**), fixed; (**c**), decalcified; and (**d**), resin-embedded transverse sections within the same bovine tarsis. Dehydrated and fixed samples yield similar THG images, while significant differences appear for decalcified and PMMA-embedded samples: in the former case, the modulation anticorrelated with the SHG signal is more pronounced and the cement line gives rise to a positive contrast. In the latter, the contrast of the canaliculi is reduced. Scale bars, 50 µm. Insets, zooms into the orange squares. Scale bars, 10 µm.

**Figure 8:**
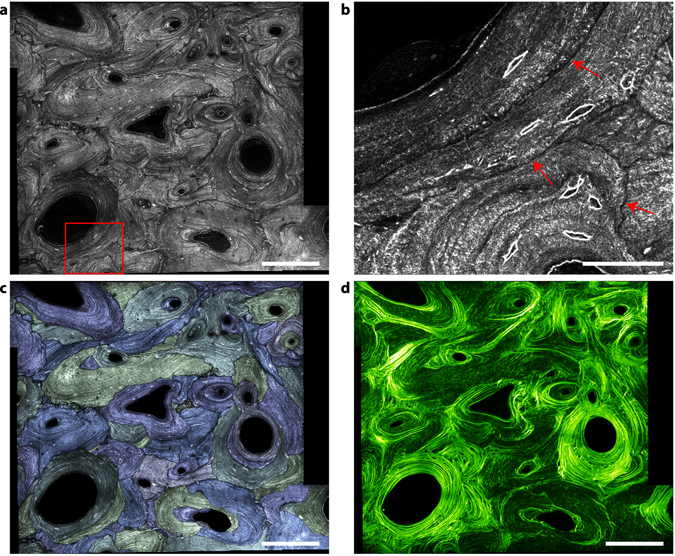
Segmentation of bone structural units using THG images. (**a**) Mosaic THG image of a human femoral cortical bone exhibiting extensive remodeling. (**b**) Zoom into the red rectangle in (**a**) showing the dip in the THG signal used to segment BSUs (red arrows). (**c**) Segmented THG with the different BSUs displayed using different colors. (**d**) Corresponding SHG image. Scale bars, 200 µm in (**a**–**c**); 50 µm in (**b**).

**Figure 9:**
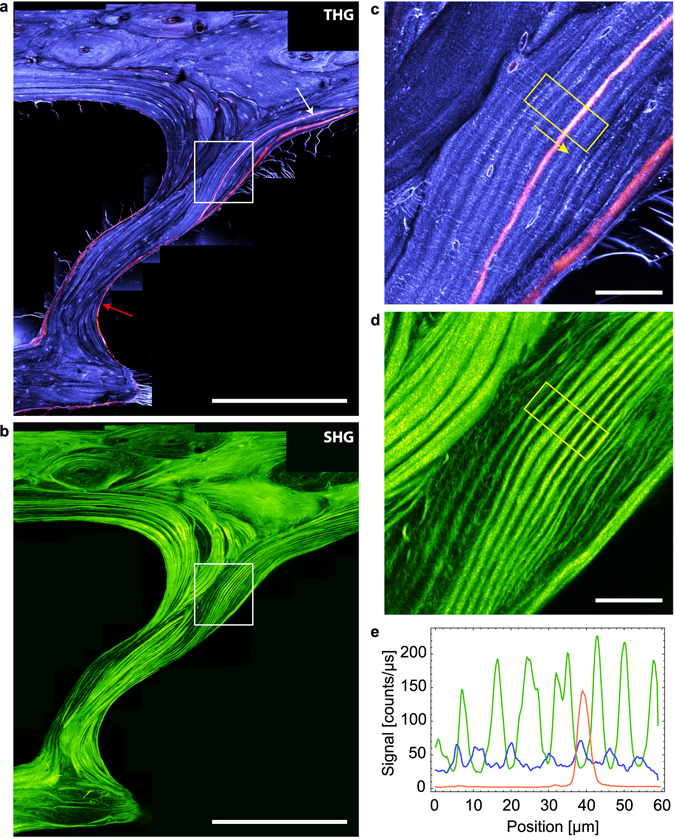
Combined fluorescence/THG/SHG imaging. THG (**a**) and SHG (**b**) images of the iliac crest of an ewe. In (**a**), THG is shown in purple and fluorescence in orange. The white arrow indicates a location where calcein was incorporating during tissue formation. Scale bars, 500 µm. (**c**) (resp. (**d)**), zoom into the white rectangle in (**a**) (resp. (**b**)). Scale bars, 50 µm. (**e**), THG (blue), SHG (green) and fluorescence (orange) profiles along the yellow box in (**c**,**d**), showing the anticorrelation between THG and SHG signals. The modulation of the THG signal is of much smaller amplitude than that of the SHG signal.

LCN analysis is generally conducted with confocal or two-photon excitation fluorescence microscopy on living, fixed or dehydrated samples using an appropriate dye which diffuses in the porosities. However, such studies are not possible on decalcified or embedded bone samples. In decalcified bone, the fluorescent dye infiltrates the whole tissue which prevents distinguishing the LCN from the surrounding tissue (see Supplementary Fig. 3c). In embedded samples, the dye simply cannot penetrate and staining must be performed prior to embedding, which leads to heavier sample preparation protocols^53^. We anticipated that THG imaging could overcome those limitations since it directly reveals endogenous optical heterogeneities. To test this idea, we prepared four adjacent sections of a bovine tarsis (cortical region) with different protocols: fresh, dehydrated, decalcified and embedded in PMMA. As shown in Fig. 7, the LCN is clearly visible in all samples. In particular, we found little difference between THG images of dehydrated (Fig. 7a) and fresh (Fig. 7b) samples. However, differences are visible in the images of the two other samples: in the decalcified sample (Fig. 7c), the modification of the composition of the matrix strongly enhances the modulation in the THG signal correlated with the collagen organization, increasing the variations both in the background and in the signal stemming from the LCN (see e.g. Fig. 7c, inset). As expected, the collagen organization revealed by the SHG signal (see Supplementary Fig. 4) is not significantly modified by the decalcification process. Interestingly, the largest change in THG contrast occurs at the cement line that, now, gives rise to a positive contrast. These changes evidence the role of the structure of the bone matrix in the coherent buildup of the THG signal. The situation is different for the resin-embedded sample (Fig. 7d). In this case, the lacunae are well delineated, possibly as a result of a well-defined resin/matrix boundary. However, the visibility of canaliculi is altered depending on their orientation. Canaliculi lying in the plane of observation appear very faint and are in some cases barely visible. On the other hand, those oriented out of plane appear as bright dots or ellipses (see also Figs 8 and 9). As a consequence, with similar imaging conditions, the visualization of the full LCN in embedded samples would require further data post-processing, as would be the case for the X-ray nanoCT data presented here, and contrary to THG images of non-embedded samples. Conversely, the THG signal modulation due to the structure of the bone matrix appears more clearly, although the modulation itself is not of greater amplitude. The strong negative contrast of the cement line, in particular, permits a straightforward segmentation of the osteon and of the bone structural units (BSU). This is demonstrated on an embedded section of human femoral cortical bone exhibiting extensive remodeling (Fig. 8): here, the THG-based domain segmentation provides information complementary to that of the matrix organization revealed by SHG imaging (Fig. 8d), which is not affected by resin embedding.

### Analyzing bone remodeling using multimodal THG, SHG and fluorescence imaging

It is of general importance to combine morphological and functional information when studying tissue development and remodeling. THG/SHG contrasts are easily combined with fluorescence imaging using multiphoton fluorescence excitation, as illustrated in Fig. 9 using a resin-embedded section of the iliac crest of an ewe. This sample was analyzed in a previous study focused on the age-dependence of the bone mineral density and mechanical properties in bone packets identified by intravital calcein and tretracyclin staining at different stages of animal growth^54^. Both fluorophores are known to have a strong affinity for calcium, such that the injection in the blood results in a strong accumulation at the mineralization front of the forming bone at the time of injection. Thus, the calcein and tetracyclin act as temporal references and any bone volume formed after the injection will be separated from the rest by a fluorescent interface. A region comprising cortical (top) and trabecular bone (bottom) is shown in Fig. 9. The limit of the trabecular bone can be identified in the SHG image as the intense, nearly continuous layer of endosteal bone forming highly regular lamellar motifs. The structure of the cortical bone is more complex and consists of volumes with high and low SHG intensity indicating variable degrees of collagen organization and orientation with respect to the observation plane.

The corresponding merged THG (purple)/fluorescence (orange) image is shown in Fig. 9a. As discussed in the previous subsection, the canaliculi are poorly visible in THG due to embedding but both the lacunae and bone matrix structures are visible. Fluorescence can be attributed to calcein fixed at the mineralization front (white arrow) which also appears to cover a part of the endost due to staining at later stages (at the trabecular surface, red arrow). The age of the region of the tissue in between the two fluorescent lines (yellow arrow in Fig. 9c) can be estimated to 12 months. No apparent differences between this volume and the adjacent tissue can be observed: THG shows that the lacunae are similar in shape, size and spatial distribution and SHG reveals a lamellar collagenous structure on both sides of the calcein boundary. Interestingly, however, the spacing between two bright layers in SHG is slightly larger in the region corresponding to the stained mineral, pointing to a local perturbation in the collagen organization on both sides of the mineralization front, as recently suggested by other structural studies^55^. This illustrates the unique capabilities of multimodal THG-SHG-2PEF imaging for structural and functional studies.

## Discussion

The results presented here demonstrate the versatility of THG as a means to characterize bone structure by revealing interfaces at different scales in a label-free manner. This might prove particularly useful in cases where sample staining is impossible or impractical as, e.g., for archeological artefacts which must be preserved, for decalcified bone sections used in medical diagnosis (e.g. anatomopathology), or for bone tissue already stained for other purposes. Furthermore, resin-embedding is an important step in the long-term conservation of histological sections. Such sample collections represent a huge potential for the analysis of the LCN which, often, cannot be performed by optical means due to the absence of staining prior to embedding. Although the THG contrast is significantly weaker in embedded samples, the canaliculi are at least partly visible and their contrast could be improved by image processing. Interestingly, the contrast of the lacunae is reinforced as compared to non-embedded samples, making THG a well-suited tool for studies focused on lacunae characterization in embedded samples.

Overall, THG provides all the advantageous features of confocal microscopy for the analysis of the LCN (large accessible field of view and straightforward 3D image reconstruction), but with additional benefits: a visualization without staining and a direct correspondence with the tissue structure as revealed by SHG. Furthermore, THG offers a complementary approach to X-ray micro/nanoCT imaging by providing access to larger areas thank to mosaicking along two dimensions. In our experimental conditions, THG imaging was practical down to typical depths of 50 µm; the implementation of X-ray nano-CT for an equivalent sub-micrometric resolution permits to explore 500 µm in depth but with a restricted section and sample size of typically 500 µm. While mosaicking might be possible along one dimension, this limits the structural information to volumes restricted in two dimensions, and increases radiation damage effects due to X-ray dose concentration (Supplementary Figure 5). Since heterogeneities in the network can occur over the scale of the whole organ, their characterization requires imaging over distances in the millimeter or even centimeter range. Thus, while X-ray nanoCT coupled to magnified phase contrast imaging offers important perspectives in terms of spatial resolution (of the order of 100 nm with an isotropic voxel size down to 20 nm^29, 48^), THG imaging enables multiscale studies of the LCN on a much larger range in two dimensions, with minimal perturbation to the sample.

Together with the segmentation of bone structural units, this permits a global study of tissue interfaces. As an example, the analysis of the whole cortical transverse section of a mouse femur with THG imaging (Fig. 4 and Supplementary Movie 5) allowed identifying qualitative differences in the LCN morphology between anatomical quadrants, as well as across the cortical section. Similarly, the LCN organization is found to differ in bovine bone according to tissue type as observed by SHG (Fig. 5 and Supplementary Movie 6). This allows analyzing the structure of the LCN in relation with that of the matrix and larger-scale structures: an example is shown in the large scale images of Figs 3 and 4 illustrating that the organization of collagen fibrils and that of the LCN are correlated in both mouse and bovine bones, as already described in a previous study^28^. We found, in particular, that well-organized collagen lamellae, which produce strong SHG modulations, are associated with lacunae elongated along the main direction of the lamellae and with canaliculi aligned perpendicular to this direction. In contrast, regions with less structured and rapidly fluctuating SHG signals (a signature of collagen fibril assemblies exhibiting disorder at scales ranging from sub-micron to tens of microns) are characterized by round lacunae with isotropically oriented canaliculi. A similar observation can be made for the lacunar morphology in human bone. The fact that this trend is verified in such extreme cases of bone formation confirms the observations of Kerschnitzki *et al*. suggesting a universal relation between collagen organization and osteocytes morphology. Similarly, combination with fluorescence imaging of specific structures opens the way to relating structural and functional information on the tissue, as illustrated in Fig. 9. It should be noted, here, that many fluorophores can be used for combined fluorescence/THG measurements using either two- or three-photon excitation^56^.

Additional information on bone structure can be extracted from combined SHG/THG images based on a precise analysis of the contrast mechanisms of both imaging methods. The origin of THG signals has been studied experimentally and theoretically for a large number of biological tissues. It is well established that THG is created in the presence of an interface or an optical heterogeneity at the scale of the focal volume (a few hundreds of nanometers and above)^15, 57–59^. This accounts for the contrasted signal highlighting canaliculi, boundaries of lacunae, vessels and Haversian channels in dehydrated, fixed and decalcified samples. In these cases the signal stems from the optical differences between the bone matrix and the mounting fluid. In the case of the resin-embedded sample, the reduction in the visibility of the canaliculi can be explained by the penetration of the resin inside the channels and possibly in the peri-canalicular tissue, resulting in an optically less contrasted interface. According to this sole hypothesis, the contrast at the border of the lacunae should also be reduced. Instead, we observe an increase in contrast. This phenomenon is likely related to a resin shrinkage, which is more pronounced within the lacunae due to their larger volume. Such a contraction would create a gap between the bone matrix and the resin, resulting in a strong THG response at the boundaries of the lacunae. Alternatively, the difference in contrast between canaliculi and lacunae could stem from a different structure or composition of the peri-lacunar and peri-canalicular bones giving rise to a sharper tissue/resin boundary in the lacunae.

The origin of the SHG signal in fibrillar collagen-rich tissues, in turn, is well established and is directly correlated with the density, alignment and orientation of the fibrils. In bone, the structure of epidetected SHG images permits discriminating between well-organized lamellar regions, where the regular modulation of the SHG signal can be linked to that of the fibril orientation with respect to the image plane^43^, and less organized areas with reduced signal variations and a shorter correlation length. In such regions, the average SHG signal is, again, related to the average orientation of the fibrils with respect to the image plane: high SHG signals correspond to fibrils tilted by a small angle with respect to the observation plane, while similarly organized fibrils at almost right angle to the observation plane produce dark regions in the SHG image (see e.g. Fig. 9b)^37, 41, 42^. Note that we also expect a modulation as a function of the in-plane angle between the collagen fibrils and the excitation polarization, but that the observed modulation is limited, probably due to geometrical effects related to out-of-plane fibril orientation and to polarization distortion in our strong focusing conditions.

Based on the anticorrelation found between the THG and epicollected SHG signals in the THG/SHG images, we attribute the origin of the THG signal arising from the bone matrix (Fig. 9e) to the following mechanism: the THG signal is likely created at the surfaces of mineralized collagen fibrils, as already described for fibrillar collagen in other tissues^19^. This mechanism is consistent with the negative contrast of the cement line, which is known to be a region devoid of collagen^9, 60^ as confirmed by our SHG images (see Supplementary Figure 6), and with the lower THG signal observed locally for dehydrated, fixed and embedded samples (Fig. 7a,b and d and Supplementary Figure 6a). In contrast, decalcification could induce a partial collapse of the cement line, resulting in a thin gap between two collagen-rich regions and producing a positive THG signal similar to that of the canaliculi (Fig. 7c). Our data and findings therefore open interesting perspectives for the study of the local variations of bone matrix properties using combined SHG/THG microscopy.

In short, we have shown that the porous lacuno-canalicular network (LCN) can be directly visualized using label-free THG microscopy in 3D with submicron precision over millimetric fields of view compatible with histology. Besides the LCN descriptions in various samples reported in this article, we have shown that THG contrast enables automated digitization of this network, opening the way to systematic large-scale studies, in particular in preparations where staining is not practical. Additionally, we find that this imaging modality also reveals interfaces delineating volumes formed at successive remodeling stages. Since THG imaging is easily combined with SHG signals mapping the bone collagen organization and with fluorescence imaging, our results demonstrate that multimodal multiphoton imaging provides a unique means to analyze the multiscale organization of interfaces in bone and their relation to tissue function in contexts such as remodeling and development.

## Methods

### Standard sample preparation protocol for optical microscopy observations

The standard procedure for sample preparation for linear and non-linear optical microscopy involved mechanical removal of soft tissue including bone marrow and fixation using ethanol 70%_v_ from 48 h for thin slices (<500 µm thick) to 10 days for cortical blocks of 10 × 10 × 10 mm^3^ and whole mouse femurs. Sections of 300 µm in thickness were cut in transverse direction using a high precision diamond saw (Mecatome T210, PRESI. France) and were further reduced by lapping on both sides with 2400 grade SiC paper (Minitec 233, PRESI, France) to 200 ± 1 µm in thickness using a calibrated stainless steel wedge. The samples were subsequently dehydrated in a graded series of ethanol solutions of 80%_v_, 90%_v_ and 100%_v_ and dried in air, between 2 glass slides in a closed petri dish, for 48 h minimum. When fluorescent staining or embedding was required, the samples were further dehydrated in vacuum for 24 h. The samples were then mounted between 2 cover slips in glycerol with a 200 µm shim surrounding the sample, and sealed with nail polish.

Polymethyl-methacrylate (PMMA) embedding was achieved according to the previously described procedure^61^.

### Samples

#### Bovine

For Figs 2–4 and 6, Supplementary Figures 2 and 5, and Supplementary Movies 1–4 and 6, a bovine femur was obtained from the local slaughter house. A 5 cm thick block of bovine bone was cut in the diaphysis and dehydrated as described above_._

For Fig. 7 and Supplementary Figures 1, 3 and 4, a fresh bovine metacarpus was collected from the slaughter house immediately following euthanasia. A transverse block of 3 cm in thickness was sewn in the mid-diaphysis. From this cortical shell, the posterior quadrant was cut in 10 × 10 × 30 mm^3^ and cut in three blocks of 10 × 10 × 10 mm^3^ with the cross-section corresponding to the transverse plane. Within 90 min following euthanasia, the central block was fixed in glutaraldehyde 4%_v_ for 48 h, rinced twice in a cacodylate buffer 0.2 M at pH 7.4 for 1 h and kept at 4 °C. A transverse section of 300 µm in thickness was cut from the central block for THG imaging. The remaining blocks were dehydrated in ethanol as described above. Two sections were cut from the dehydrated upper block adjacent to the central fixed one. One was measured as such and the other was decalcified using a solution of EDTA 16%_V_ at pH 8.6 for 72 h by replacing with fresh solution every 2 h during the first 24 h and once a day subsequently. Finally, the remaining lower block was embedded in PMMA.

#### Mouse

A 12-week old C57B16 mouse was dissected immediately following euthanasia. A femur was dehydrated in ethanol as described above. To facilitate manipulation, the sample was embedded in epoxy resin (Epofix, Struers) using a 15:2 resin/hardener ratio which only adheres to the periost with limited penetration inside the porosity. A 300 µm thick section was, then, cut from the mid-diaphysis. All studies were ethically reviewed and carried out in accordance with European Directives 2010/63/UE on the care, welfare and treatment of animals. All the procedures were reviewed by the ethics committee affiliated to the animal facility of the university (D3842110001).

#### Ewe

An iliac crest biopsy was removed from an ewe (6 year-old) of a study on the time sequence of secondary mineralization^54^. Bone sample was fixed in 70% ethanol and embedded in PMMA^61^.

#### Human

The anterior quadrant of a cross-section (10 mm in thickness) was obtained from the femoral midshaft of a 92-year-old female cadaver. Ethical approval for collection of samples was granted by the Human Ethics Committee of the Centre du don des corps at the University Paris Descartes (Paris, France). The tissue donors or their legal guardians provided informed written consent to give their tissue for investigation, in accord with legal clauses stated in the French Code of Public Health. The sample was chemically fixed for ten days in 70% ethanol, then dehydrated in 100% ethanol and embedded in PMMA. All methods were performed in accordance with the relevant guidelines and regulations.

### Fluorescence staining

Staining was performed with FITC at 0.1%_W_ in glycerol for 72 h and sample were mounted in the staining medium, except for X-ray nanoCT comparison for which ethanol was used instead of glycerol.

### Multimodal imaging comparison

For site-matched comparison between THG, CSLM and X-ray nanoCT, a previously prepared 200 µm thin section was fixed with double-sided tape on a counter block of PMMA and cut in 400 µm wide stripes extending through the whole cortical shell from the endost and to the periost.

The samples were, first, stained with FITC and imaged using confocal microscopy followed by combined THG, 3PEF and SHG. The samples were then unmounted and left in ethanol 100%_v_ to remove the dye excess and dried in vacuum for 72 h before performing the X-ray nanoCT experiments. In order to assess the effect of radiation damage of the X-ray experiment on the sample ultrastructure, the samples were also imaged consecutively to X-ray exposure by confocal and THG/SHG using the same instruments and staining protocols as described above.

### Multiphoton imaging

Imaging was performed on a custom-built laser scanning microscope incorporating a femtosecond infrared source (KTP OPO (APE, Germany) pumped by a Ti:sapphire laser (Coherent, USA) or Insight DS + (Newport SpectraPhysics, USA)), galvanometer mirrors (GSI Lumonics, USA) and near-infrared objectives (Olympus, Japan). A water-immersion objective (UPLSApo 60XW, 1.2NA) was used in all experiments, except for Fig. 1b for which an oil-immersion, high NA objective was used (UPLSApo 60XO, 1.35NA). Excitation wavelength was 1.18 µm. Excitation power was adjusted as a function of depth in the range 40–150 mW using a motorized wave plate and a polarizer, pulse duration at the sample was 100fs, and pixel dwell time was in the range 5–20 μs depending on the experiment. Beam polarization was linear. Signals were detected using photomultiplier modules (SensTech, UK), and FPGA-based lab-designed counting electronics. Scanning and acquisition were synchronized using lab-written LabVIEW software and a multichannel I/O board (PCI-6115, National Instruments, USA). THG was detected in the forward direction through a bandpass filter (FF01-377/50-25 filter, Semrock, USA), while SHG and fluorescence were epidetected using a dichroic mirror (695dcxru, Chroma, USA) and directed toward two independent detectors using additional dichroic mirrors and filters (FF560-FDi01dichroic mirror, Semrock, USA; for fluorescence, e700sp short-pass filter, Chroma; for Fig. 8, a FF01-525/50-25 (Semrock) was added; For SHG, FF01-590/20-25 filter, Semrock, USA). Theoretical and experimental values of lateral and axial resolutions are given in Supplementary Table 1.

### Confocal microscopy

Confocal imaging was performed on a motorized inverted confocal microscope (SP8, Leica) using a 40x, 1.3NA oil objective. FITC was excited at 488 nm and the fluorescence was collected in the 500–600 nm range on a hybrid detector. Brightfield transmission images were acquired simultaneously using the same excitation beam. Excitation power at the focal point was adjusted as a function of depth in the range 6–70 µW. Pixel dwell time was 1.8µs. The theoretical resolution in the absence of aberrations was 230 nm (lateral) and 1.0 µm (axial).

#### Synchrotron nano-CT

Bone sections were imaged using Synchrotron Radiation (SR) nano-CT at ID19 of the ESRF (European Synchrotron Radiation Facility, Grenoble, France) with 280 nm isotopic voxel size^35^. For each sample, 2499 projection images were recorded over a total angular range of 360° at a fixed energy of 19 keV. A 4.8 μm thick LSO scintillator was used to transform X-rays into visible light which was detected with an E2V CCD camera. The total time for each scan was around 35 minutes. A field of view about 0.57 × 0.57 × 0.57 mm^3^ was scanned. The acquisitions were processed using a standard 3D-filtered backprojection algorithm to get the 3D reconstructed images. 3D Gaussian filtering with standard deviation of one pixel was applied to reduce noise.

### Data analysis

Data analysis was performed using ImageJ, Mathematica and home-written macros. Registration of the confocal, μSR-CT and nonlinear images in Fig. 1 was achieved through manually determined rigid rotations of the 3D volumes, and the subsequent compensation of the field deformations using plugin BUnwrapJ^62^.

For segmentation of the LCN, images were acquired using a 200 × 200 × 200 nm^3^ voxel size. The contrast of confocal images was first enhanced by non linear filtering using a Vesselness filter (ImageJ plugin Frangi’s vesselness, see ref. 63 for details), thresholded and subsequently skeletonized using ImageJ plugin skeletonize^64^, providing a 3D map of the canaliculi. Lacunae were segmented by applying a threshold on the raw images, followed by a series of open/close operations to remove the canaliculi. For THG images, a first step of deconvolution of the 3D images was necessary to improve the contrast of images. An effective 3D point spread function (PSF) was estimated from the transverse sections of canaliculi in the stack of images that were analyzed. Deconvolution was performed with Mathematica using this experimental effective PSF and a Richardson-Lucy algorithm. Deconvolved images were then skeletonized after thresholding.

Segmentation of the BSUs was performed on images with a pixel size of 200 × 200 nm^2^ using a combination of manual thresholding and skeletonization, and manual correction and completion of the segmenting lines.

The signal-to-background ratio of the different imaging modalities was estimated by comparing the average signal in the center of the canaliculi to the one measured at least 1 µm away from any structure in the sample. For nanoCT images, because the LCN provides a negative contrast, the inverse ratio was used for comparison.

Correlation coefficient between two images are calculated using the following formula:

1 $$c({I}_{1},{I}_{2})=\frac{\langle ({I}_{1}-\langle {I}_{1}\rangle )({I}_{2}-\langle {I}_{2}\rangle )\rangle }{\sqrt{\langle {({I}_{1}-\langle {I}_{1}\rangle )}^{2}\rangle \langle {({I}_{2}-\langle {I}_{2}\rangle )}^{2}\rangle }},$$

where the brackets denote averaging over the image. The coefficient is 1 for perfect correlation, −1 for perfect anti-correlation, and 0 in the absence of correlation between the two images I_1_ and I_2_.

## Electronic supplementary material

Supplementary information file

Combined THG/fluorescence imaging of the LCN at high resolution.

Combined THG/fluorescence imaging of the LCN at the cement line.

Comparison of different bone imaging modalities.

Comparison of different bone imaging modalities.

Imaging mouse bone structures at the organ scale.

Imaging structural variability in bovine bone.

Combined fluorescence/THG/SHG imaging.
