# Supplementary material for: Label-free imaging of bone multiscale porosity and interfaces using third-harmonic generation microscopy
Source: Sci Rep. 2017 Jun 13;7:3419. doi: 10.1038/s41598-017-03548-5 (PMC5469828; doi:10.1038/s41598-017-03548-5)
Supplement: Supplementary file 1 — Supplementary information file [file 41598_2017_3548_MOESM1_ESM.pdf]

Label-free imaging of bone multiscale porosity and interfaces using third-harmonic generation microscopy.

Rachel Genthial[1,2], Emmanuel Beaurepaire[3], Marie-Claire Schanne-Klein[3], Françoise Peyrin[4,5], Delphine Farlay[6,7], Cécile Olivier[4,5], Yohann Bala[6,7], Georges Boivin[6,7], Jean-Claude Vial[1,2], Delphine Débarre[1,2*], Aurélien Gourrier[1,2]

[1] Univ. Grenoble Alpes, LIPHY, F-38000 Grenoble, France
[2] CNRS, LIPHY, F-38000 Grenoble, France
[3] LOB, Ecole Polytechnique, CNRS, Inserm, Université Paris-Saclay, F-91120 Palaiseau, France
[4] Université de Lyon, CREATIS, CNRS UMR5220, Inserm U1206, INSA-Lyon, Université Claude Bernard Lyon 1, France
[5] ESRF, European Synchrotron Radiation Facility, F-38000, Grenoble, France
[6] INSERM, UMR 1033, F-69008 LYON, France
[7] Université de Lyon, F-69008 LYON, France

*Corresponding author: delphine.debarre@univ-grenoble-alpes.fr

Supplementary Information

		Theory	THG	Fluorescence
NA=1.2	lateral	0.32 µm	0.43 µm	0.41 µm
	axial	0.90 µm	1.8 µm	1.75 µm
NA=1.35	lateral	0.29 µm	0.35 µm	0.34 µm
	axial	0.74 µm	1.35 µm	1.3 µm

Table S1: theoretical and experimental values of lateral and axial resolutions in THG and multiphoton fluorescence. Although a point-spread function cannot be defined, strictly speaking, for THG imaging, the resolution can be estimated theoretically by assuming that they are comparable to that of three-photon excited fluorescence imaging at the same wavelength. When using a high NA objective and in the absence of aberration, an estimate for the lateral (FWHMx) and axial (FWHMz) resolution can be inferred from Zipfel et al.[1]. Experimental measurements were measured on the dataset shown in movie S2 and S3. Fluorescence FWHMs were computed from intensity profiles by taking into account the width of the canaliculi (as measured with THG, see Fig. 1). Fluorescence and THG measured FWHMs are similar, and significantly larger than theoretical values. This is consistent with the assumption that relatively large aberrations are created when focusing in or close to canaliculi, as inferred from lateral THG profiles on these structures.

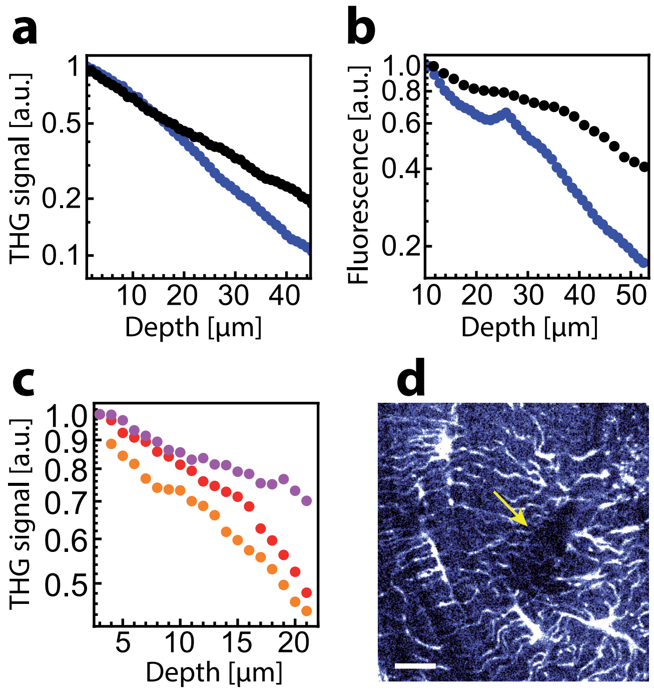

Supplementary figure 1: Typical penetration depths in THG and confocal microscopy. Signal depth profiles acquired in bovine samples with various preparations. The loss of signal with depth is due both to the losses of excitation intensity due to scattering, and to aberrations that distort the focal spot. a (resp. b), THG (resp. confocal fluorescence) signal attenuation as a function of the immersion medium. Attenuation in glycerol (black) is less pronounced than in water (blue), allowing deeper imaging. We attribute this effect to the reduction of refractive index mismatch between the bone matrix and the porosities that distorts the focal spot. The effect is more severe in the case of THG imaging because of the highly nonlinear dependence to the focal spot intensity and despite the larger excitation wavelength that usually reduces the strength of scattering within biological tissues. c, comparison of THG signal attenuation for fixed (orange), dehydrated (red) and embedded (purple) samples. Again, the filling of the porosities with embedding resin has a positive impact on the penetration depth, probably due to a reduction of the refractive index mismatch. d, example of reduced THG signal (arrow) observed when imaging through a lacuna in a dehydrated, ethanol-embedded sample. Such shadows degrade significantly the quality of imaging at depths greater than a few tens of microns. Scale bar, 10µm.

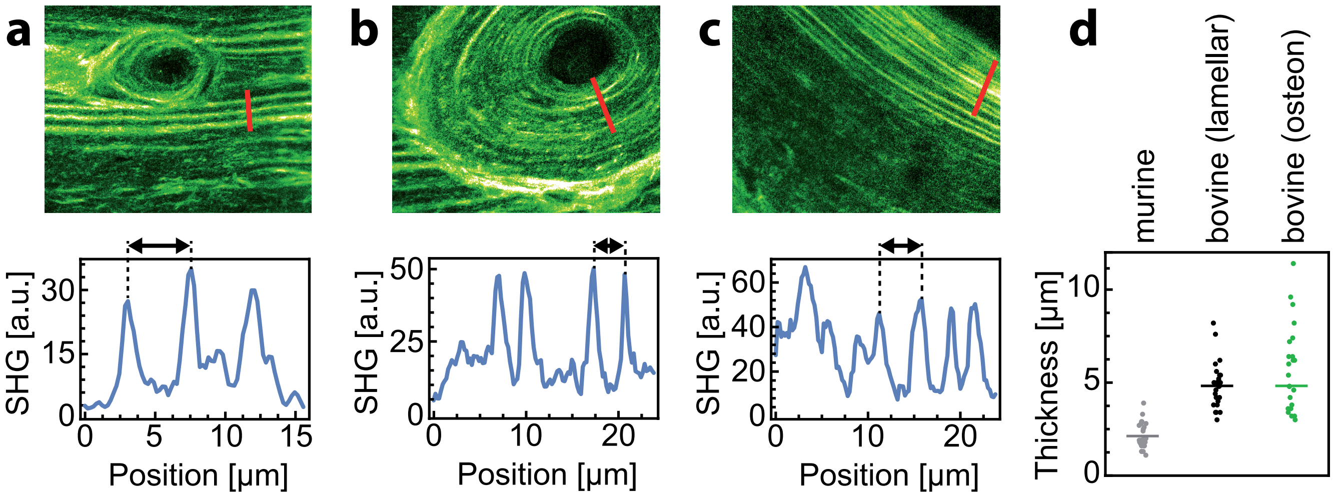

Supplementary figure 2: Quantification of the thickness of lamellae in mouse and bovine samples. SHG profiles were plotted at various locations in each sample. For each profile, thicknesses of lamellae were measured as the distance between consecutive signal maxima. Examples of profiles are shown for bovine lamellar bone (a), osteon (b) and for murine bone near the endost (c). All samples are transverse sections of dehydrated femurs. d, average thicknesses of lamellae were calculated from ~25 measurements for each sample. The thickness of lamellae appears significantly larger in the bovine femur, although more statistics on several animals should be used to confirm this observation.

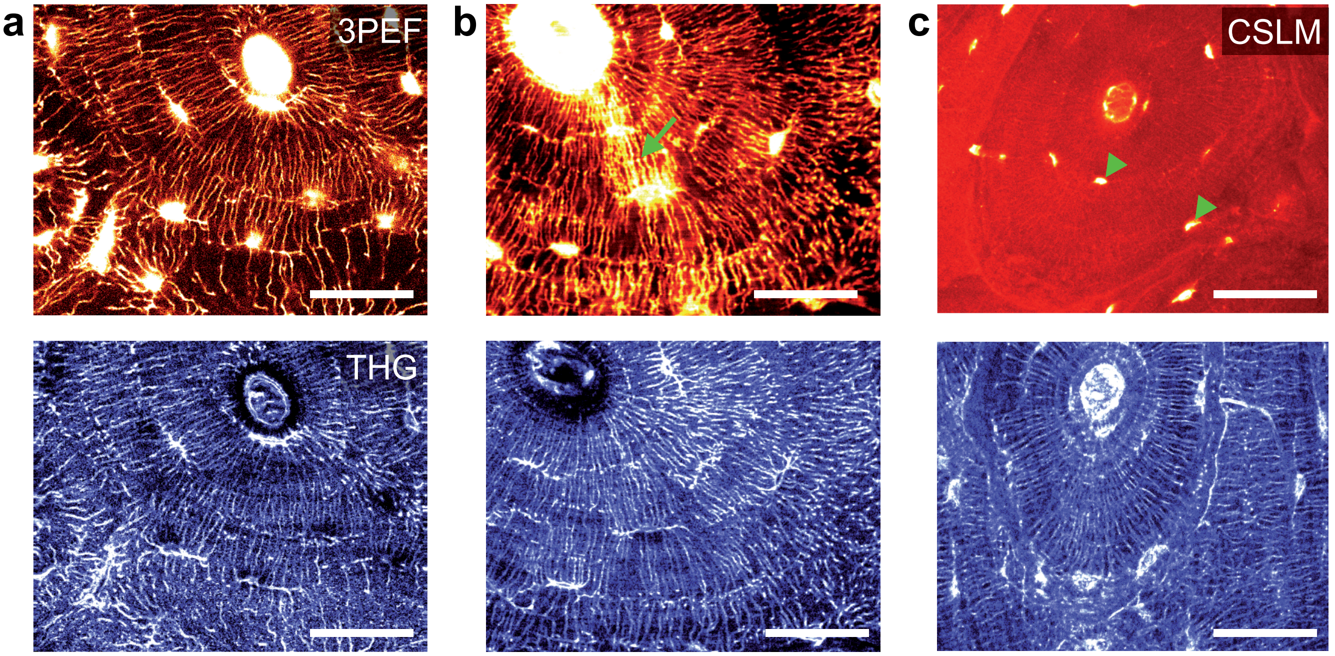

Supplementary figure 3: Influence of the sample preparation on fluorescence images. In degreased samples (a), both the fluorescent (3PEF, top) and the THG (bottom) signals nicely delineate the network. In fixed samples (b), the fluorescent staining seems to penetrate within the bone matrix in places (arrow), while the THG signal is still well defined. In decalcified samples (c), the staining (visualized using CLSM) permeates the matrix to such extend that only the lacunae remain visible (arrowheads), while canaliculi are still clearly visible in THG images. Scale bar, 50µm.

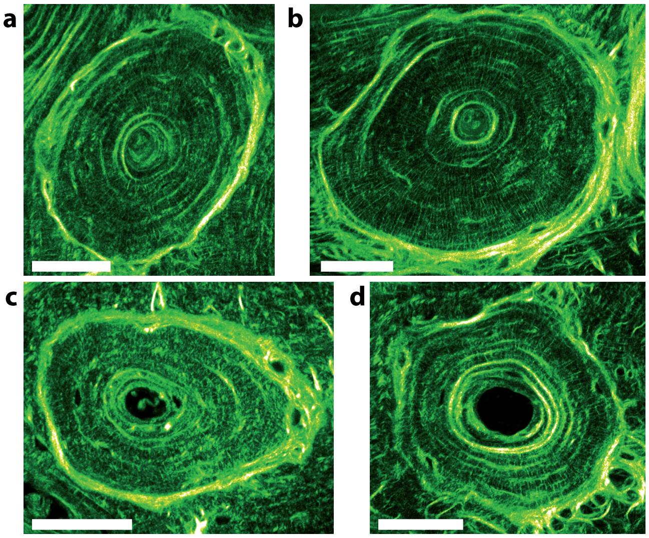

Supplementary figure 4: Influence of the sample preparation on SHG images. The SHG images correspond to the same fields of view as the THG images in Fig. 7. No significant change in the SHG signal is observed beyond the osteon-to-osteon variability. Signal inside the Haversian channel most probably stems from imperfectly suppressed fluorescence from residual organic material. Scale bars, 50 μm.

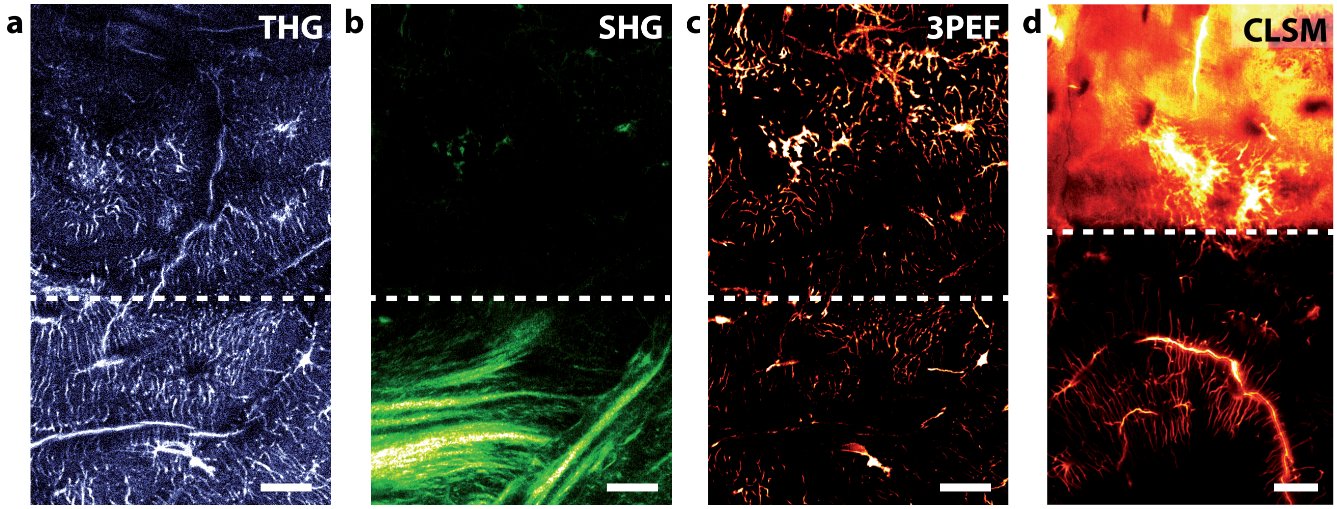

Supplementary figure 5: Effect of X ray nano-CT imaging on bone matrix structure imaged with combined THG/SHG/fluorescence. Significant changes in the structure of the sample can be detected after nano-CT imaging (above the white dotted lines). THG signal is reduced (a), while the SHG signal from organized collagen fibers completely disappears, indicating extended damage to the organic matrix. This correlated with an increased penetration of the dye within the matrix (c, three-photon excited fluorescence), which in some cases can lead to an ubiquitous fluorescent signal throughout the sample (d). The last image was acquired separately with confocal microscopy (CLSM) on another sample stained with aqueous Rhodamine B. Increased penetration might be partly linked to the difference in the dye, but is most probably mainly due to a longer exposure to X-rays during alignment. Scale bars, 20 μm.

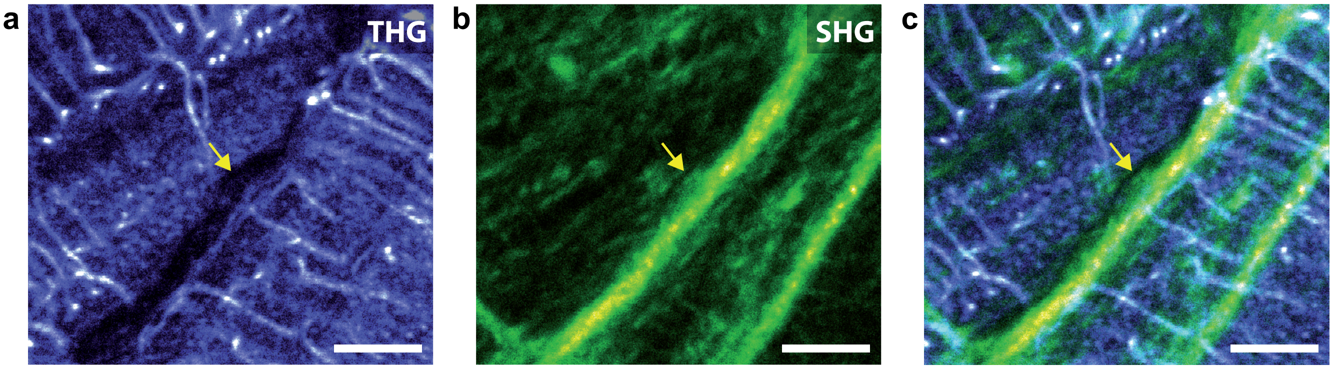

Supplementary figure 6: THG/SHG imaging of the cement line. a, high resolution THG image of the cement line between lamellar tissue (left) and an osteon (right). The cement line appears as a darker region that in places extend towards the inner of the osteon. b, corresponding SHG image showing a narrow dip in the SHG signal at the position of the cement line. A region with strong SHG signal is frequently observed in the close vicinity of this line toward the center of the osteon. c, composite THG (blue)/SHG (green) image illustrating the negative contrast of the cement line for both modalities. Scale bar, 10µm.

Supplementary Movie 1: Combined THG/fluorescence imaging of the LCN at high resolution. z-stack of a transverse section of degreased bovine femur imaged with THG (left, purple) and multiphoton fluorescence (right, orange) using the 1.35NA, oil objective. The sample and imaged area are the same as in Fig. 1b. Comparison between the two images confirms that THG permits visualizing the canaliculi. With such high NA objective, the two edges of the canaliculi are visible, thus allowing to measure their diameter (see Fig. 1e). Spacing between two successive planes, 300nm. Scale bar, 10 µm.

Supplementary Movie 2: Combined THG/fluorescence imaging of the LCN at the cement line. z-stack of a transverse section of degreased bovine femur imaged with THG (left, purple) and multiphoton fluorescence (right, orange) using the 1.2NA, oil objective. The sample is the same as in Supplementary Movie S1 and Fig. 1 but the imaged area is distant by a few hundred microns. The strong fluorescence signal at the top of the stack is due to the accumulating dye at the surface of the sample. The cement line, lying horizontally at about half way up the image, is most visible on the THG image and separates an older osteon (bottom part) from a more recent one (upper part). Note the presence of several canaliculi crossing the cement line, the abrupt ending of most canaliculi at the cement line on the side of the older osteon, and the change in direction of canaliculi along the cement line in the newer one. Such dataset provides invaluable information on the influence of remodeling on the local structure of the network. Spacing between two successive planes, 300nm. Scale bar, 10 µm.

Supplementary Movie 3: Comparison of different bone imaging modalities. z-stack of the full field images of Fig. 3a, showing a transverse section of a bovine femur imaged with THG (top left), 3PEF (top middle), SHG (top right), fluorescence confocal (CLSM, bottom left), brightfield transmission (BF, bottom middle) and X-ray nanoCT (bottom right). THG, 3PEF and SHG images were acquired simultaneously. CLSM and BF images were recorded next, followed by X-ray nanoCT images. The three sets of images were registered manually. Distance between two successive planes, 200nm. Scale bar, 50 µm.

Supplementary Movie 4: Comparison of different bone imaging modalities. z-stack of the zoomed images of Fig. 3b, showing a transverse section of a bovine femur imaged with THG (top left), 3PEF (top middle), SHG (top right), fluorescence confocal (CLSM, bottom left), brightfield transmission (BF, bottom middle) and X-ray nanoCT (bottom right). THG, 3PEF and SHG images were acquired simultaneously. CLSM and BF images were recorded next, followed by X-ray nanoCT images. The three sets of images were registered manually. Distance between two successive planes, 200nm. Scale bar, 10 μm.

Supplementary Movie 5: Imaging mouse bone structures at the organ scale. Whole section of a mouse femur imaged with THG (left) and SHG (right). The data are the same as in Figure 5. Scale bar, 100 μm. The inset shows the position of the zoom (red square) on the whole transverse femoral section.

Supplementary Movie 6: Imaging structural variability in bovine bone. Large-scale image of a transverse section of cortical bovine femur in THG (left) and SHG (right). The data are presented in Figure 7. This large-scale image includes an osteon with a well-defined radial organization (around the Haversian channel, red arrow) and a surrounding fibrolamellar region. Scale bar, 50 μm.

Supplementary Movie 7: Combined fluorescence/THG/SHG imaging. THG (left) and SHG (right) images of the iliac crest of an ewe. The data are presented in Figure 9 with the addition of multiphoton fluorescence. Scale bars, 100 μm.

References

1. Zipfel, W. R., Williams, R. M., Webb, W. W. Nonlinear magic: multiphoton microscopy in the biosciences. *Nat. Biotechnol.* **21**, 1369–1377 (2003).
